# Associations among Genotype 1b Hepatitis C Virus Core Protein, Protein Kinase R, and Signal Transducer and Activator of Transcription 3

**Published:** 2010-12-01

**Authors:** Xue bing Yan, Zhi Chen, Christian Brechot

**Affiliations:** 1 Department of Infectious Diseases, The First Affiliated Hospital of Xuzhou Medical College, Xuzhou 221002, Jiangsu Province, China; 2 Institute of Infectious Diseases, the First Affiliated Hospital, College of Medical Science, Key Laboratory of Ministry Health, Zhejian; 3 University, Hangzhou 310003, Zhejiang Province, China

**Keywords:** Hepatitis C virus, Core protein, Protein kinase R, Signal transducer, transcription 3

## Abstract

**Background and Aims:**

Because hepatitis C virus (HCV) core protein (Core), protein kinase R (PKR), and signal transducer and activator of transcription 3 (STAT3) all play relevant roles in the pathogenesis of HCV, persistent infection and hepatocellular carcinoma (HCC) and PKR may interact with HCV Core. In this study, we further investigate the associations among HCV Core, PKR, and STAT3 and the mechanisms involved in these interactions.

**Materials and Methods:**

Expression levels of HCV Core, PKR, eukaryotic initiation factor 2 (eIF-2α), phosphorylated eIF- 2α (p-eIF-2α), STAT3, and phosphorylated-STAT3 (p-STAT3) were compared between Huh-7 and replicon cell-Huh-7 cells harboring the full length of genotype 1b HCV genomes. Co-immunoprecipitation and glutathione S-transferase (GST) pull-down assay were conducted for HCV Core, PKR, and STAT3.

**Results:**

HCV may have induced the expression of STAT3 and the activity of PKR (p-eIF-2α). HCV Core, STAT3, and PKR appear to have interacted with one another. The N-terminal 1-126 amino acid (aa) of HCV Core contributed to an interaction between HCV Core and STAT3, and only full-length PKR bound to STAT3 and p-STAT3.

**Conclusions:**

These findings suggest that HCV Core, PKR, and STAT3 can interact with each other. Specifically, HCV Core may play its role through both PKR and STAT3. Alternatively, HCV Core’s binding to and activation of STAT3 might be due to the interaction between HCV Core and PKR. The distinct interactions among these three molecules are important and may reveal a new molecular mechanism in the pathogenesis of HCV-persistent infection and HCV-related HCC.

## Intrudction

Hepatitis C virus (HCV) often causes a prolonged and persistent infection and is associated with hepatocellular carcinoma (HCC) [1][2]. The pathogenesis of liver damage is at least in part related to viral protein-mediated factors. Understanding the molecular basis of pathogenesis is a major challenge in gaining insight into HCV-associated disease progression. Recent experimental evidence using HCV-cloned genomic regions suggests that the genotype 1b HCV core protein (HCV Core) has numerous functional activities [3][4]. These include HCV Core’s likely role in regulatory effects on cellular and unrelated viral promoters, interactions with a number of cellular proteins, a modulatory role in programmed cell death or apoptosis under certain conditions, involvement in cell-growth promotion and immortalization, induction of HCC in transgenic mice, and a possible immunoregulatory role. These intriguing properties suggest that HCV Core may contribute to pathogenesis during persistent infection and HCC, but the exact mechanisms of HCV remains unclear [5]. Of the many proteins activated by HCV replication, both interferon-induced, double-stranded, RNA-activated protein kinase R (PKR) and signal transducer and activator of transcription 3 (STAT3) are the subjects of considerable attention [6][7][8]. PKR is a key arm of the antiviral and antiproliferative effects of interferon. PKR binds to dsRNA, resulting in a conformational change that leads to autophosphorylation on several serine and threonine residues. PKR then dimerizes and phosphorylates serine residue 51 on the alpha-subunit of the eukaryotic initiation factor 2 (eIF-2a). Phosphorylated eIF-2α (p-eIF-2α) inhibits translation initiation and decreases the rate of protein synthesis [6][7]. STAT-family proteins are transcription factors critical in mediating cellular signalling. Among them, STAT3 is often constitutively phosphorylated (p-STAT3) in human cancers and implicated in tumorigenesis. Constitutive activation of STAT3 and PKR have been demonstrated to be associated with malignant transformation induced by various oncoprotein. Different domains of PKR and STAT3 might have different functions. Both PKR and STAT3 affect normal cellular functions, such as cell proliferation, death and play an important role in cell transformation and tumors [6][7][8], PKR and STAT3 are both present in greater number in HCV-derived HCC tumor tissue than nontumor tissue [9][10]. PKR might be necessary for STAT3 phosphorylation after platelet-derived growth factor (PDGF) stimulation [11]. Because HCV Core not only binds to and interacts with PKR [12] but also cooperates with STAT3 and leads to cellular transformation [8], PKR and STAT3 may therefore contribute to HCV’s viral persistence and its association with HCC. Our previous studies found that HCV Core can interact with PKR [12] and that the interactions might be a general phenomenon, regardless of HCV genotype and strain. Based on these findings, we examine whether or not HCV Core can directly interact with STAT3, PKR, with STAT3, and the mechanisms by which these interactions occur. We further demonstrate the direct interactions among HCV Core, PKR, and STAT3 and propose two models of associations between the three in the pathogenesis of persistent HCV and HCC.

## Materals And Methods

### Plasmid construction

**Figure 1:**
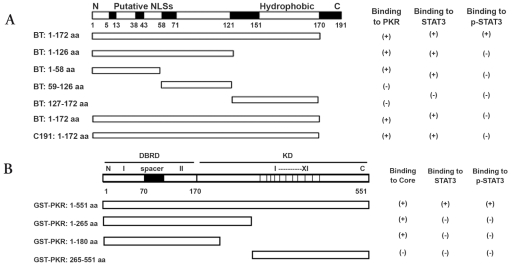
Schematic representation of the open reading frame encoding various truncated Core and PKR. A: BT: Core gene from tumor tissue of the patient with HCC caused by HCV; BNT: Core gene from non-tumor tissue of the patient with HCC caused by HCV; C191: Core gene from genotype 1b, HCV-J6. B: DRBD: double stranded RNA binding domain, KD: kinase domain. Functional domains are shown in shadowed boxes along the diagrams. Results of the binding experiments are shown on the right.

Different glutathione--transferase (GST) Core or Core-truncated fusion proteins were constructed using the pGEX-4T-1vector. The different core sequences were amplified from the pDP18 vector containing tumor (T) or non-tumor (NT) Core sequences from patient B (BT and BNT) in Delhem et al. [6]. or HCV core genotype 1b, C191, isolated from serum (Figure 1A). The following primers were designed according to the different core sequences and the various sizes desired. Plasmid construction was conducted according to YAN et al. [12] PCR products were cloned at the EcoR-I and BamH-I sites in the pGEX-4T-1 vector to obtain various GST-core fusion proteins. Full-length and different truncated GST-PKR expression plasmids were generous gifts from Dr. Meurs (Pasteur Institute, France) according to [13] full-length PKR corresponded to amino acid (aa) 1–551, and truncated forms to PKR aa 1–265, PKR aa 1–180, and PKR aa 265–551 Figure 1B.

### Cell culture

The human hepatoma cell lines HepG-2 and Huh-7 were grown in Dulbecco’s modified eagle medium (DMEM, Invitrogen, France) supplemented with 10% foetal bovine serum (FBS, Invitrogen, France), 2 mM L-glutamine, 100 U/ml penicillin, 100 µg/ml streptomycin, and 2.5 µg/ml plasmocin. Cells from the full-length HCV replicon cell line (A kind gift from C. Rice, Rockefeller University, New York City, USA), were cultured in DMEM containing 10% FBS, 100 U/ml penicillin, 100 µg/ml streptomycin, and G418 250 µg/ml. For the cell-protein extracts, about 2×106 cells were seeded in 10-cm dishes, and 24 hours after plating, stimulated with human recombinant interferon-alpha-2α 500 U/ml for 18 hours (R&D Europe, France). Cells were lysed in 300 µl cell-lysis buffer (20 mM Tris pH 7.8, 150 mM NaCl, 0.5 mM EDTA, 0.5 mM PMSF, NP40 0.2%) and frozen at -80°C for 15 minutes. Homogenates were centrifuged at 12,000 rpm for 5 min. Proteins were quantified with a Beckman 600 using the Bio-Rad protein assay (Bio-Rad, France).

### SDS-PAGE and Western blotting

50 µg of total protein, co-immunoprecipitation, and GST pull-down assay elution buffer were loaded onto an SDS 10% polyacrylamid gel, transferred onto a 0.22-nm nitrocellulose membrane, and blotted using different first antibodies: anti-STAT3, anti-p-STAT3, anti-PKR, anti-eIF-2α, anti-p-eIF-2α (1/1000: Santa Cruz, Biotechnology, USA), anti-HCV Core, and second antibodies. Blots were incubated with the peroxydase-coupled second antibodies anti-mouse: 1/2000 and anti-rabbit: 1/5000 (Amsersham, Pharmacia, USA), and proteins were visualized using a chemiluminescence detection kit (ECL, Amersham, Pharmacia, USA).

### Co-immunoprecipitation analysis

Total proteins of 500 µg were precleared at 4°c for 1 hour with 20 µl of protein G-sepharose 4 fast-flow beads (Amsershan; Biosciences) and then centrifuged at 15,000 rpm for 5 minutes. After preclearing, the supernatant was incubated in cell-lysis buffer overnight at 4°c under gentle rotation with polyclonal anti-PKR (1/100), anti-STAT3 (1/300), and 30 µl of protein G. The beads were washed 3 times with TBS buffer (50 mmol/L Tris pH 7.6, 150 mmol/L NaCl, 0.5% triton) and solubilized in 2xSDS-PAGE loading buffer and centrifuged at 1,500 rpm. As described above, the supernatant was subjected to a western blot.

### Production of GST fusion proteins

GST-core and GST-PKR fusion proteins were expressed in Escherichia coli BL21 (ED3), harboring the respective plasmids as described by Bonnet et al. [14].Cells were grown overnight at 37°C in 100 ml terrific broth (TB) containing 100 µg/ml of ampicillin. A 1/100th volume of overnight culture was inoculated in 100 ml fresh medium, grown at 30°C to an OD of 0.5 at 600 nm and then induced with 0.5 mM isopropyl-1-thiod-galactopyranoside (IPTG) for exactly 2 hours. Cells were harvested by centrifugation at 4,000 rpm for 10 minutes at 4°C, resuspended in 5 ml prechilled PBS containing 0.2 mM PMSF and 1 mM DTT, then frozen and thawed twice at -80°C. After 4 sonications of 20 seconds each on ice, Triton X-100 was added at a final concentration of 1%. Lysates were centrifuged at 9,000 rpm for 20 minutes at 4°C. Supernatants (5 ml) were incubated with 250 µl glutathion-sepharose-4B beads (Amersham, France) for 1 hour at 4°C under gentle rotation. After centrifugation at 5,000 rpm for 5 min, the beads were washed 4 times with PBS and protease inhibitors, suspended in 2.5 ml 30% glycerol-PBS and stored at -80°C. The concentration of GST-fusion proteins was estimated by comparison with a known concentration of BSA using SDS-PAGE with Coomassie Blue Staining (Bio-safe, Bio-Rad).

### GST pull-down assay

1 µg of different truncated GST-PKR fusion proteins were used per pull-down assay. GST-4B empty beads were used to adjust each reaction system with all 30 µl of beads. The beads were incubated with 120 µg of protein extraction in a final volume of 200 µl of HNGT (20 mmol/L HEPES pH 7,9, 150 mmol/L NaCl, 10% glycerol, 0.1% Triton X-100, 0,2 mmol/L PMSF, 1 mmol/L DTT) buffer overnight at 4°C under gentle rotation. Following incubation, the reaction mixtures were centrifuged at 1,500 rpm for 5 minutes at 4°C and washed 5 times with 1 ml of cold binding buffer of HNGT. After a final wash, proteins bound to the beads were eluted by boiling in a 2×SDS-PAGE sample buffer. After centrifugation, the supernatants were subjected to SDS-PAGE and immunoblotted.

## Results

### HCV increases the expression levels of STAT3 and p-eIF-2α

**Figure 2:**
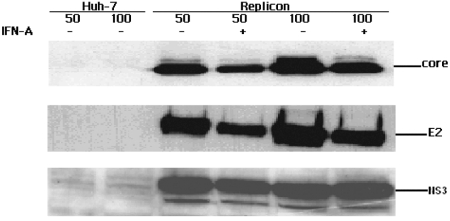
Expression levels of different HCV proteins induced with IFN-α. Using western blot analysis, different proteins including Core, E2 and NS3 were expressed in Replicon cell. Expression levels of the different proteins were suppressed after IFN-α stimulation. 50, 100 stand for input of 50 μg and 100 μg cell protein.

**Figure 3:**
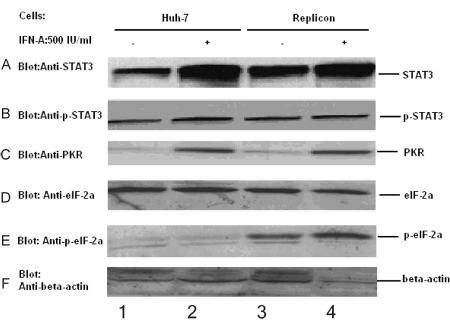
HCV induced the expression of STAT3, p-STAT3, PKR, eIF-2α and p-eIF-2α 50 μg of total cell extract was used to western blot. Cells were treated with IFN-α 500 IU/ml for 18 hours. Stimulation with IFN-α increased the expression level of STAT3 and PKR in both Huh-7 and replicon (Figure.2: A, C: lane 2, 4 to lane 1, 3), but without change in the level of p-STAT3, eIF-2α and p-eIF-2α (Figure.2: B, D, E). Both with and without IFN-α stimulation STAT3 and p-eIF-2α is higher in replicon compared with in Huh-7 (Figure.2: E lane 3,4 to lane 1,2), but there is no difference in p-STAT3, PKR and eIF-2α between Huh-7 and replicon both with and without IFN-α stimulation (Figure.2: A, B, C, D: lane 1, 2 to lane 3, 4).

**Figure 7:**
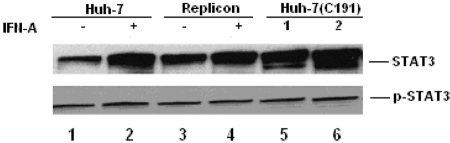
GST pull-down assay among HCV Core, PKR, STAT3 and p-STAT3. In Replicon and Huh-7 cells, GST-Core 1-172 aa (A: lane 2) and GST-PKR 1-551 aa (A: lane 3) could bind with STAT3. GST-PKR (B: lane 3; D, E: lane 2) could bind with p-STAT3 but not the GST-Core. Only full-length of GST-PKR (1-551)(C, D, E: lane 2) bound with STAT3 and p-STAT3 both in Huh-7 and replicon cells with or without IFN-α stimulation

We first conducted a western blot to find if all HCV proteins were expressed in replicon cells (Huh-7 cell line harboring selective full-length of HCV genomic). As shown in figure 2, structure protein E2, HCV Core, and nonstructure protein NS3 of HCV were all expressed in replicon cells. After stimulation with IFN-α, levels of E2, HCV Core, and NS3 were decreased, which indicated that IFN-a could suppress the replication of replicon cells and expression of E2, HCV Core, and NS3. Then replicon cells and Huh-7 cells were used to observe if HCV could affect the expression of STAT3, p-STAT3, PKR, eIF-2α, and p-eIF-2α, respectively. eIF-2α and p-eIF-2α were markers of PKR activity. Stimulation with IFN-α resulted in an increase in the induction of PKR and STAT3 in both Huh-7 and replicon (Figures 3A and 3C, lanes 2 and 4 to lanes 1 and 3, respectively), but without change in the level of p-STAT3, eIF-2α and p-eIF-2α (Figures 3B, 3D, and 3E). Both with and without IFN-α stimulation STAT3 and p-eIF-2α is higher in replicon compared with Huh-7 Figure 3E,lanes 3 and 4 to lanes 1 and 2, but there is no difference in p-STAT3, PKR, and eIF-2α between Huh-7 and replicon both with and without IFN-α stimulation (Figures 3B,3C and 3D lane 1 and 2 to lane 3 and 4), which suggests that HCV could affect the STAT3 expression and activity of PKR, but not the expression levels of PKR and p-STAT3. The results were further confirmed by next experiment with PKR, STAT3, and p-STAT3 (Figures 7C,7D, and 7E).

### HCV Core induces the expression of STAT3

**Figure 4:**
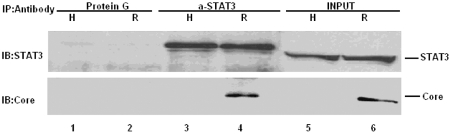
STAT3 and p-STAT3 expression in different cells: Huh-7: 1-2; replicon: lane 3-4; Huh-7 transfected with HCV Core from C191 with 1 and 2 ug of plasmids respectively lane 5-6. 2, 4 was stimulated with IFN-α 500 IU/ml for 18 hours; lane 1, 3, 5, 6 without IFN-α stimulation.

As shown above, HCV could increase the STAT3 expression and activity of PKR, but not the expression levels of PKR and p-STAT3. Because replicon cells can express E2, HCV Core, and NS3, we conducted another experiment to investigate whether HCV Core could increase the expression of STAT3 and PKR. In the same cell line (Huh-7), Huh-7 transfected with plasmid containing HCV Core (C191) and Huh-7 harboring selective full-length HCV genome (replicon), and different expression levels of STAT3 were found (Figure 4). The level of STAT3 in transfected cells was higher compared with the levels in Huh-7 and replicon, and the STAT3 level in replicon was higher than that of Huh-7; however, there was no obvious difference in p-STAT3 among them. These findings indicate that HCV could affect the STAT3 expression, mainly due to HCV Core. In a previous study, we found that HCV Core could increase the activity of PKR [6][12].

### HCV Core physically interacts with STAT3

**Figure 5:**
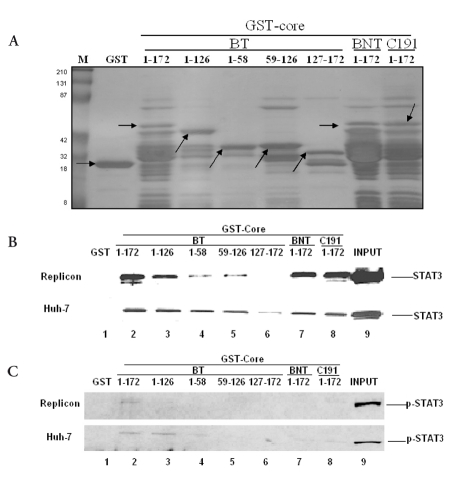
HCV Core physically interacts with STAT3: Whole cell lysates (500 μg) from Huh-7 (H: lane 1, 3, 5), replicon cells (R: lane 2, 4, 6) were used for immunoprecipitation with polyclonal anti-STAT3 (Santa Cruz). Immunoprecipitates were separated by SDS-PAGE and transfered to nitrocellulor membranes and immunoblotted with anti-STAT3 (upper panel) and anti-Core (lower panel). Lane 1, 2: protein G without antibody served as negative control. Lane 5, 6: 1/10 input (50 μg of cell protein).

**Figure 6:**
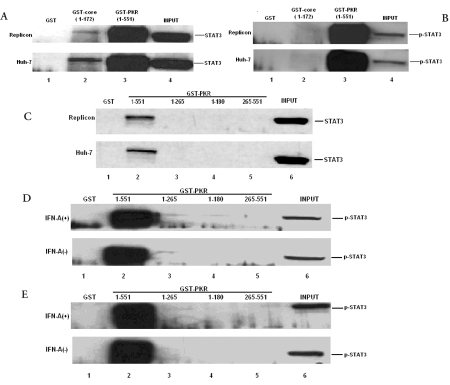
The N-terminus of Core (aa 1-126) is involved in the interaction with STAT3 A: In order to verify in each GST pull-down assay reaction system approximately equal amount of GST-Core was used, 1 μg of different truncated GST-Core was used to do Coomassie Blue Staining. M: marker; Lane 1: GST, Lane 2-6: different truncated GST-Core of BT; Lane 7: BNT; Lane 8: C191. Arrow stands for target bands. B, C: Control GST (lane 1) and GST fused to different truncated Core (lanes 3 to 8) were incubated in the presence of in vitro expressed STAT3 and p-STAT3 in different cells(replicon, Huh-7) (lanes 2- 8). Lane 9, 50% input ( 60 μg of total protein).

To determine whether STAT3 is present in a complex with HCV Core, a co-immunoprecipitation assay was performed with anti-STAT3 polyclonal antibody. The results indicated that HCV Core and STAT3 were physically associated (Figure 5). This specific interaction between HCV Core and STAT3 was further demonstrated by in vitro GST pull-down (Figures 6B7A and 7B). GST fused to the HCV Core 1-172 amino acid (aa) of BT, BNT, and C191 (but not the control GST), and clearly pulled down in vitro-expressed STAT3 in different cells (Huh-7, replicon;(Figure 6B). These results strongly suggest that HCV Core forms a complex with STAT3.

### The N-terminus of HCV Core aa 1-126 including the two fragments 1-58 aa and 59-126 aa is involved in the interaction with STAT3

In order to map which domain was responsible for the HCV Core/STAT3 and HCV Core/p-STAT3 interactions, a GST pull-down assay was performed. As shown in figure 6B, there was no binding band in lane 1 (GST), suggesting that the HCV Core/STAT3 binding was specific. GST-Core 1-58, GST-Core 59-126, GST-Core 1-126, and GST-Core 1-172 (BT, BNT, C191), but not the control GST (GST-Core 127-172), expressed STAT3 when pulled down in vitro. These results suggest that the smallest binding domains are 1-58 aa and 59-126 aa of HCV Core. The binding affinity decreased as the fusion proteins (BT: 1-172 aa, 1-126 aa, 1-58 aa, 59-126 aa) became shorter, which indicates that the neighboring flanking region of N-terminal 1-58 aa and 59-126 aa might also contribute to efficient binding or that one of the two fragments enhances the other’s binding affinity. There is some difference of HCV Core/STAT3 and HCV Core/p-STAT3 binding affinity among the different HCV strains with the same length 1-172 aa (BT, BNT, C191). The binding between HCV Core from BT and STAT3 (Figure 6B and lane 2) is stronger than that the binding between HCV Core from BNT and C191 (Figure 6B and6C, lanes 7 and 8), and only HCV Core from BT can bind to p-STAT3 (Figure 6C). The result was further verified by repeating the experiment (Figure 7).

### PKR physically interacts with STAT3 and only full-length PKR binds with STAT3 and p-STAT3

To determine the region of PKR responsible for the interaction between PKR and STAT3, a GST pull-down assay was performed, using different truncated GST-PKR. As shown in figures 7C, 7D, and 7E, only full-length PKR, and not the control GST, GST-PKR 1-265 aa, GST-PKR 1-180 aa, or GST-PKR 265-551 aa pulled down with STAT3 and p-STAT3 in different cell lines (Huh-7, replicon) with and without INF-α stimulation. The result suggests that the binding between PKR and STAT3 might depend on the conformation of PKR. The binding intensity of PKR and STAT3 and the domain of PKR between Huh-7 and replicon with and without INF-α stimulation was almost identical, which suggests that the PKR and STAT3 binding might also be independent of HCV. When performing the PKR and p-STAT3 GST pull-down assay, we found that p-STAT3 was higher bound with PKR than STAT3 both in Huh-7 and replicon with and without INF-α stimulation, which indicates that PKR might induce STAT3 phosphorylation and that direct interaction between PKR and STAT  might increase the activation of STAT3.

## Discussion

**Figure 8:**
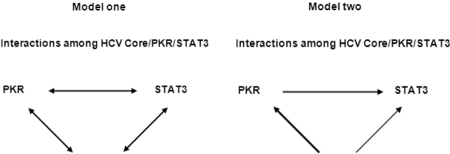
Models of interactions among HCV Core, PKR and STAT3. Model one: HCV Core, PKR and STAT3 can physically directly interact with each other and might form a ternary complex. Model two: HCV Core plays its function through both of PKR and STAT3 but mainly through PKR, HCV Core binding to STAT3 and activating STAT3 might be due to interaction or activation of Core/ PKR.

HCV Core has been extensively studied and appears to play multiple roles in various cellular signaling pathways, potentially even in oncogenesis. Different domains of HCV Core might have various functions [15]. Versatile functions of HCV Core have been mapped to its N-terminal half. HCV Core is hydrophilic and contains clusters of basic aa residues, which serve as putative localization signals (NLS) as well as binding domains for RNA and ribosome [16]. Moreover, the N-terminal region of HCV Core has been shown to be involved in its multimerization, transcriptional regulation, and interaction with certain cellular factors, including lymphotoxin-beta receptor, heterognous nuclear ribonucleo-protein K, and RNA helicase [17][18][19][20]. We found that the interaction between HCV Core and PKR depended on N-terminal 1-58 aa of HCV Core and N-terminal 1-180 aa of PKR [12], which might increase the translocation of PKR from cytopalsmic to nuclear. It is possible that, in HCV-infected individuals, different truncated HCV Core  might exist, with shorter HCV Core, more in the nuclear region. Our findings confirm the results of another recent study, which found that nuclear HCV Core facilitated the translocation of PKR into nucleoli where the two proteins colocalized are suggestive of a direct interaction between truncated HCV Core and PKR. Nucleolar translocation of PKR might indeed reflect its activation [21]. In this work, we further demonstrated that N-terminal 1-126 aa of HCV Core can directly bind to STAT3. The HCV genome exhibits a considerable degree of variation and is currently classified into 6 genotypes and more than 60 subtypes [22][23]. Even with the same subtype, there exist quasi-species. Quasi-species constitute a pool of viral variants that can change and acquire new selective advantages in a very short time; thus, these new variants have adaptive advantages with a modified viral tropism, host range, virulence, and drug resistance. Therefore, Patients infected with different HCV genotypes might have unique clinical features [24], and different HCV genotypes might contribute some of their own characteristics in the pathogenesis. HCV Core could induce the expression of STAT3, and HCV Core from BT, BNT and C191 (they all belong to HCV genotype 1b) in vitro can bind to STAT3. Furthermore, only HCV Core from BT could bind to p-STAT3, which might be one of the characteristics of HCV genotype 1b in its pathogenesis, or at least one pathogenesis characteristic of some HCV genotype 1b strains, and which might account for why incidences of liver cirrhosis and HCC are higher in HCV genotype 1b than those of other genotypes. Because we did not construct HCV Core expression plasmids of other HCV genotypes, so we cannot generalize our conclusion to all other HCV genotypes. However, HCV Core encoding genome among different genotypes is highly conserved. Comparing the nucleotide and aa sequences of BT, BNT, and C191, they contain high homology at aa and nucleotide levels [6]. Additionally, BT did not contain a specific motif; therefore, the interaction between HCV Core and STAT3 might be genotype independent. One study found that a heterologous overexpression of HCV structural and nonstructural proteins may have stronger oncogenic potential than the expression of individual viral proteins [25]. STAT3 activation can also occur with HCV Core and with the expression of whole HCV proteins. Recently, a study noted a similar constitutive activation of STAT3 by the nonstructural HCV protein NS5A [26]. But in another study, no effects of these agents on HCV-Core-mediated STAT3 activation was observed [8]. Transcriptional activity of STAT3 was also more strongly up-regulated by the full HCV genome than by HCV Core alone [8]. In our study, it is interesting that HCV Core alone more strongly induced STAT3 as compared with the whole HCV genome expression. This suggests that STAT3 may be induced and activated by multiple mechanisms and by different viral proteins. Further study is needed to define the relationship between STAT3 activation, transformation  and full HCV protein expression. HCV Core cooperates with STAT3 in malignant transformation as other viral oncoproteins or cellular protooncogenes do, thereby contributing to malignant transformation. As cells expressing HCV Core alone were not tumorigenic, for the appearance of HCC, some secondary “hit(s),” such as elevated expression of STAT3 or activation of ras [27], must be necessary for the development of a malignant phenotype. Up-regulation of cyclin D1, C-myc, and Bcl-XL may explain the increase in growth rate by activation of STAT3 [28][29], yet these two genes are not sufficient for cellular transformation. Yoshikawa and colleagues reported evidence that inactivation of SOCS1 by gene methylation and the consequent constitutive activation of cytokine-JAK/STAT (especially STAT3) signaling pathways are responsible for growth of HCCs [30]. Given that Core expression occurred only in the presence of HCV (the HCV genome is not integrated into hepatocyte DNA), the interaction between HCV Core and STAT3 may be an initial step of STAT3 activation, and then the loss of SOCS-1 expression takes over the constitutive activation of STAT3 [30]. Therefore, the constitutive activation of STAT3 induced by viral or cellular oncogenes or the loss of suppressors present an attractive target for drug discovery with the potential of inducing cell death or inhibiting the growth of human HCV-mediated hepatocarcinomas. In this study, we found that PKR bound with STAT3 and that only full-length PKR could interact with STAT3, which might depend on the conformation of PKR. The PKR/STAT3 binding intensity and domain of PKR between Huh-7 and replicon, and between PKR with and without IFN-a stimulation is almost the same, which suggests that the interaction between PKR and STAT3 might also be HCV independent. Based on our study, we proposed two models about the associations of HCV Core, PKR, and STAT3. Model 1 (as shown in Figure 8A) indicates that all 3 molecules can interact with each other. HCV Core plays its role mainly by interacting with cellular proteins. HCV Core, PKR and STAT3 might all interact together, or HCV Core might affect cell proliferation through both PKR and STAT3. It is possible that the physical association of these molecules modulates their respective activates. Two mechanisms may account for their interactions. First, heterodimerization could confer unique properties on either STAT3 or HCV Core at the level of protein stabilization or cellular localization. Second, because the HCV Core protein has no tyrosine kinase activity [17], the binding between HCV Core, PKR and STAT3 might hamper the phosphatases approach to STAT3 and PKR or directly block the phosphatic site of STAT3 and PKR and HCV Core, which eventually would cause their constitutive expression and activiation. HCV Core can induce expression of STAT3 and p-eIF-a and bind to STAT3 and some quasi-species, and HCV Core can also bind to p-STAT3. It remains unclear whether HCV Core can directly bind to and inhibit the phosphorylation of STAT3 and PKR. The manner in which HCV Core induces the tyrosine phosphorylation of STAT3 and PKR is also unclear. Another possibility is that the interaction between HCV Core and STAT3 can recruit a tyrosine kinase to STAT3. As for model 2 (Figure 8B), HCV Core’s binding to STAT3 or activation of STAT3 might be due to the interaction between or activation of HCV Core and PKR. In other words, the interaction between HCV Core and PKR might activate and increase the activity of PKR [6][12], which would inhibit HCV replication and increase cell apoptosis. PKR, as a protein [14], can act as a mediator and link other proteins. PKR can also act as an adaptor, recruiting STAT kinases through its preassociation with STAT3. Additionally, PKR is necessary for the phosphorylation of STAT3 [11][31][32], allowing for a rapid response to various stimuli. Several oncogenic proteins, including HCV Core, have been reported to activate STAT3, which might occur through PKR. HCV Core binds to PKR, although the interaction between HCV Core and PKR might induce other pathways, such as an interaction between PKR and STAT3 or an interaction between HCV Core and STAT3. PKR, as a kinase, can phosphorylate other proteins, including STAT3. Constitutive expressed PKR, by phosphorylation of C-jun to suppress the cell reaction to TGF-ß [33], leads to vicious cell generation. At the same time, PKR can act as an up-regulator in the activation of STAT3, resulting in the rapid overproliferation and vicious malignancy transformation of cells, and as a common mediator of both growth-promoting and growth-inhibitory signals. It is possible that the elevated levels of PKR result in STAT3 misregulation, even though HCV-Core-induced apoptosis of infected hepatocytes through PKR may be important in virion dissemination and in liver damage during chronic infection. It has been proposed that the proapoptotic effect of HCV Core could contribute to the development of HCC by promoting continuous regeneration of hepatic cells [21][34][35]. Although we have not been able to demonstrate a significant effect of the interactions between HCV Core, PKR, and STAT3 on cell growth, it is possible that cells resistant to HCV-Core-mediated apoptosis could have enhanced growth and a replicative cycle. This explanation is consistent with the demonstration of an accelerated cell cycle coexisting with apoptosis induction in cell clones expressing HCV Core [36][37]. We are not able to simply generalize our conclusions to physiological infection conditions. Therefore, confirmation of our hypothesis by using human specimens infected with HCV is necessary in future studies. Howe ever, this study raises a plausible mechanisms to explain interactions between HCV Core, PKR, and STAT3 and provides new insight into the mechanisms of HCV-persistent infection and HCC development. Finally, the study adds a new aspect of virological function of HCV Core. The two models might represent another viral and cellular protein interaction and might pave the way to finding a new therapy for HCV. This research Supported by The Nature Science Foundation of Jiangsu Province, No. BK2007031; The Nature Science Foundation of Xuzhou, No. XZ07C055 and 2008 Qing Lan Project of Jiangsu Province.
